# Familial Associations of Adiposity: Findings from a Cross-Sectional Study of 12,181 Parental-Offspring Trios from Belarus

**DOI:** 10.1371/journal.pone.0014607

**Published:** 2011-01-27

**Authors:** Rita Patel, Richard M. Martin, Michael S. Kramer, Emily Oken, Natalia Bogdanovich, Lidia Matush, George Davey Smith, Debbie A. Lawlor

**Affiliations:** 1 School of Social and Community Medicine, University of Bristol, Bristol, United Kingdom; 2 Medical Research Council Centre for Causal Analyses in Translational Epidemiology, University of Bristol, Bristol, United Kingdom; 3 Department of Pediatrics, McGill University Faculty of Medicine, Montreal, Canada; 4 Department of Epidemiology and Biostatistics, McGill University Faculty of Medicine, Montreal, Canada; 5 Department of Population Medicine, Harvard Medical School and Harvard Pilgrim Health Care Institute, Boston, United States of America; 6 The National Research and Applied Medicine Mother and Child Centre, Minsk, Belarus; Institute of Preventive Medicine, Denmark

## Abstract

**Background:**

It is suggested that maternal adiposity has a stronger association with offspring adiposity than does paternal adiposity. Furthermore, a recent small study reported gender assortment in parental-offspring adiposity associations. We aimed to examine these associations in one of the largest studies to date using data from a low-middle income country that has recently undergone a major political and economic transition.

**Methods and Principal Findings:**

In a cross-sectional study of 12,181 parental-offspring trios from Belarus (mean age (SD) of mothers 31.7 (4.9), fathers 34.1 (5.1) and children 6.6 (0.3) at time of assessment), we found positive graded associations of mother's and father's BMI with offspring adiposity. There was no evidence that these associations differed between mothers and fathers. For example, the odds ratio of offspring overweight or obesity (based on BMI) comparing obese and overweight mothers to normal weight mothers was 2.03 (95%CI 1.77, 2.31) in fully adjusted models; the equivalent result for father's overweight/obesity was 1.81 (1.58, 2.07). Equivalent results for offspring being in the top 10% waist circumference were 1.91 (1.67, 2.18) comparing obese/overweight to normal weight mothers and 1.72 (1.53, 1.95) comparing obese/overweight to normal weight fathers. Similarly, results for offspring being in the top 10% of percent fat mass were 1.58 (1.36, 1.84) and 1.76 (1.49, 2.07), for mother's and father's obese/overweight exposures respectively. There was no strong or consistent evidence of gender assortment - i.e. associations of maternal adiposity exposures with offspring outcomes were similar in magnitude for their daughters compared to equivalent associations in their sons and paternal associations were also similar in sons and daughters.

**Conclusions/Significance:**

These findings suggest that genetic and/or shared familial environment explain family clustering of adiposity. Interventions aimed at changing overall family lifestyle are likely to be important for population level obesity prevention.

## Introduction

One might anticipate that maternal-offspring adiposity associations would be stronger than paternal-offspring adiposity associations. Mothers may have a greater influence on their offspring's diet and other behaviours than do fathers. For example, in a UK birth cohort stronger maternal-offspring than paternal-offspring associations for fat and protein intake were reported.[1] In addition developmental overnutrition, via intrauterine mechanisms or postnally, via breastfeeding, might lead to stronger maternal-offspring associations.[2]–[5]

Results from previous studies in this area have reported inconsistencies, with some claiming stronger maternal-offspring BMI associations [6]–[10] but others showing similar magnitudes of associations between parents.[11]–[14] The majority of these studies have included fewer than 4000 parental offspring trios, none has examined associations with offspring waist circumference and fat mass, and few have explored whether findings are influenced by possible non-paternity.

A recent UK study of 226 parent-offspring trios reported marked differences in maternal-daughter compared to paternal-son adiposity associations, with daughters only being affected by their mother's obesity and son's by their fathers.[15] The authors concluded that these findings demonstrated that genetic inheritance played little role in within family clustering of obesity. However, findings from this small study require replication.

Understanding intergenerational patterns of familial clustering will help to elucidate underlying mechanisms for this clustering and hence inform preventive strategies for reducing obesity.[16]; [17] For example, if such analyses were to suggest the main driver were maternal characteristics then interventions aimed at behaviour change in women in early and mid adult life might be of particular importance. On the other hand if associations are similar for both parents and not assorted by gender than whole family based interventions are likely to be more appropriate.

The aim of this study is to add to current evidence in this area by examining parental-offspring BMI associations in a large cohort of over 12,000 parent-offspring trios from Belarus. The large sample size allows us to examine associations across the entire BMI distribution as well as the extremes of overweight/obesity. In addition, in this study we examine parental BMI in relation to offspring waist circumference and subscapular and triceps skinfold thicknesses.

## Methods

### Participants

The study sample is nested within a cluster-randomized controlled trial located in the Republic of Belarus, in which the experimental intervention was promotion of breastfeeding modelled on the WHO/UNICEF Baby-Friendly Hospital Initiative.[18] A detailed description of the original trial, called the Promotion of Breastfeeding Intervention Trial (PROBIT), has been published previously.[18] In the trial 17,046 mother-infant pairs were recruited during their postpartum hospital stay. Inclusion criteria specified that infants were full-term singleton births, weighing at least 2500 g and mothers had no illnesses that would contraindicate breast feeding and initiated breastfeeding when they entered the postpartum ward immediately after delivery. Recruitment occurred between June 1996-December 1997 at 31 hospital sites, and 97% of the trial participants were followed for the first year of life at 31 polyclinics, one affiliated to each hospital. Between December 2002 and April 2005, when the children were aged on average 6.5 years, they were invited to a follow-up interview and examination; 13,889 (81.5%) children were examined at the 31 polyclinics by one of 38 trained study pediatricians.[19]; [20]

In this paper we use information on the child's anthropometric measurements that were obtained at the 6.5 year follow-up, and the parents' height and weight, which were reported by the parent attending that clinic with the child. A total of 1,708 trios were excluded because of missing data on anthropometry/age for child (n = 17), mother (n = 161) or father (n = 1,655). The remaining 12,181 trios form the participants included in the analyses presented here.

### Measurements

#### Anthropometric measurements

At the follow-up examination, the following anthropometric measures were taken on the child in duplicate (see reference[19] for details): standing height; weight; waist circumference; subscapular and triceps skinfold thickness. The mean of the duplicate readings was used in all analyses.

At the same research visit, the parent who accompanied the child reported height and weight for both her/himself and the other parent (or responded that they did not know). For the vast majority of children (92%) the mother reported height and weight for both herself and the child's father; in a minority, the father (5%) or another guardian (2%) reported for both parents.

#### Covariables

At the birth of the study child, the location of the polyclinic was recorded as in an urban or rural location. The mother reported both parents' age at the birth of the child, number of older siblings, maternal smoking during pregnancy and both parents' occupations and education levels. Head of household occupational social class was based on whichever parent had the highest occupation and was categorised as either manual (i.e. manual worker/farmer) or non-manual (i.e. service) worker, the latter being considered the highest social class. Education was coded separately for each parent as: initial, incomplete or common secondary only; advanced secondary or partial university; and completed university. Maternal smoking in pregnancy was recoded from five categories (none, 1–4, 5–9, 10–19 and ≥20 cigarettes per day) to a binary variable (yes/no to smoking during pregnancy). At the follow-up interview, at 6.5 years, parental smoking status was recorded and classified as a binary variable as in mother's smoking status during pregnancy. Since only 2% of women reported smoking during pregnancy, smoking status for mothers at follow-up was used in all analyses; repeating analyses with the 2% of women who smoked in pregnancy removed did not alter any findings. The number of younger siblings since the birth of the study child (0, 1, 2+) was also recorded at the follow-up clinic.

The institutional ethical review board of the Montreal Children's Hospital approved the study and participating mothers gave signed consent in Russian before the examinations.

### Statistical Analyses

Percentage body fatness in children was derived using the equations of Slaughter et al from the subscapular and triceps skinfolds.[21] In order to compare associations of maternal and paternal BMI with offspring outcomes and also to compare associations with different measurements of adiposity in the offspring, we generated internally (to this cohort) age- (in 6-month categories for the offspring and 1 year categories for parents) and sex-standardized z-scores for all anthropometric measurements. Childhood BMI categories (underweight, normal weight, overweight or obese) were derived using the age- and gender-specific International Obesity Task Force (IOTF) cutpoints.[22]; [23] Parental BMI was similarly categorised. Due to small numbers, we collapsed the categories for both parents and offspring into underweight/normal versus overweight/obese. For other offspring outcomes – waist circumference, skinfolds, percent body fat – we generated binary outcomes that compared those that were in the top 10% of the distribution (i.e. at or above the 90^th^ percentile standardised for gender and age) to those below this percentile.

Age-standardized Pearson's partial correlation coefficients between all parental and offspring anthropometric measurements were calculated separately for female and male offspring. Initial multivariable regression analyses were conducted separately on female and male offspring and gender differences assessed by including an interaction term between parental BMI and offspring gender and computing a likelihood ratio test for interaction. With few exceptions there was no strong statistical evidence that associations differed by offspring gender in either parent, and results stratified by offspring gender largely looked very similar. Therefore, for the main analyses we present associations with both female and male offspring combined in the tables and discuss and provide results in the text on small number of times when there was evidence of differences by gender.

**Figure 1:**
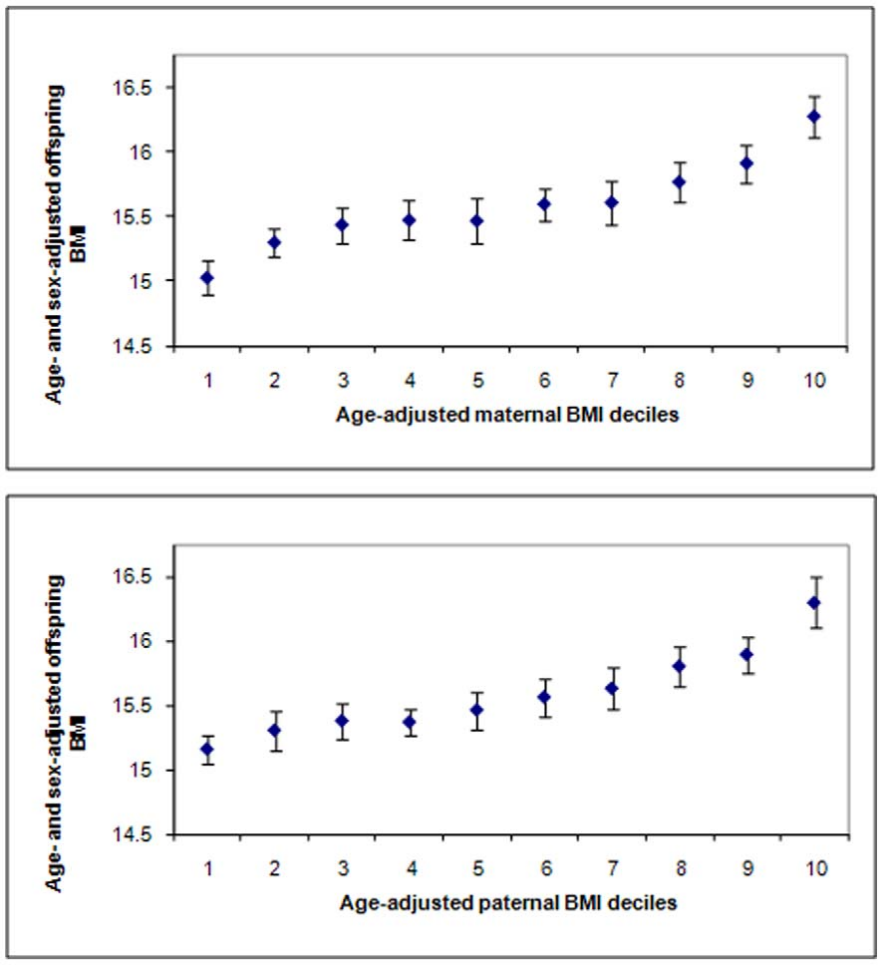
Offspring body mass index (BMI) according to deciles of maternal (top panel) and paternal (bottom panel) BMI (based on 12,181 parent-offspring pairs available). Values are mean and 95% CIs.

**Figure 2 pone-0014607-g002:**
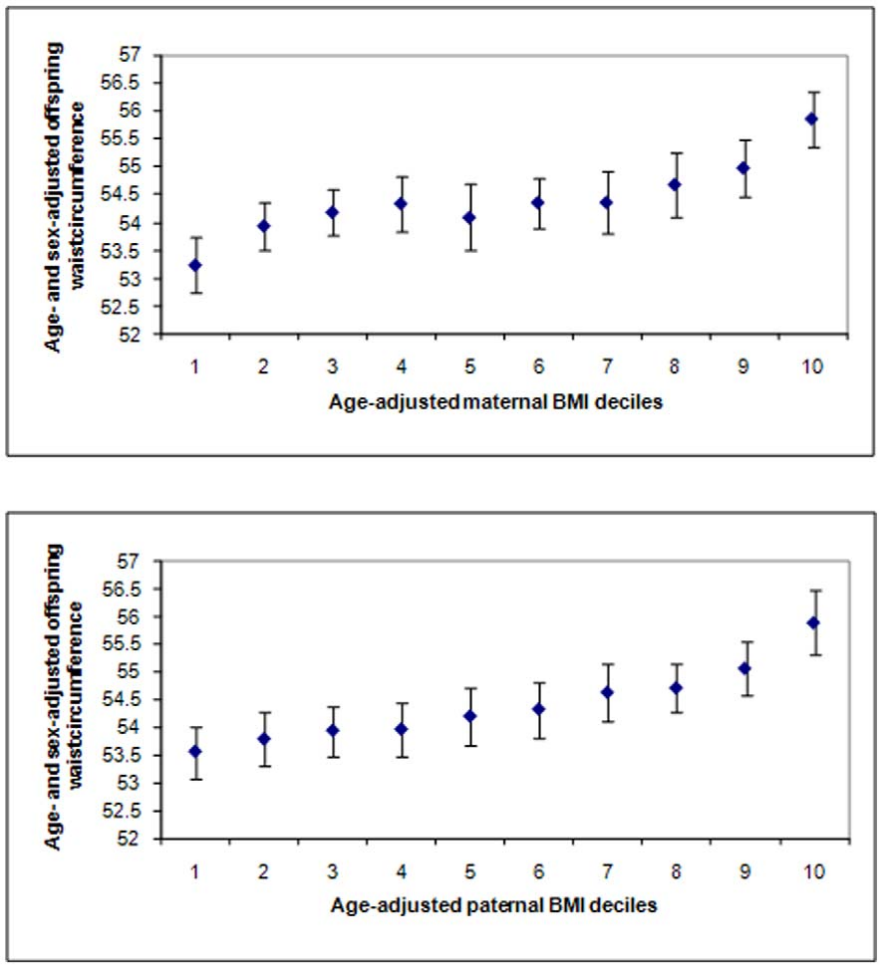
Offspring waist circumference according to deciles of maternal (top panel) and paternal (bottom panel) body mass index BMI (based on 12,181 parent-offspring pairs available). Values are mean and 95% CIs.

**Figure 3:**
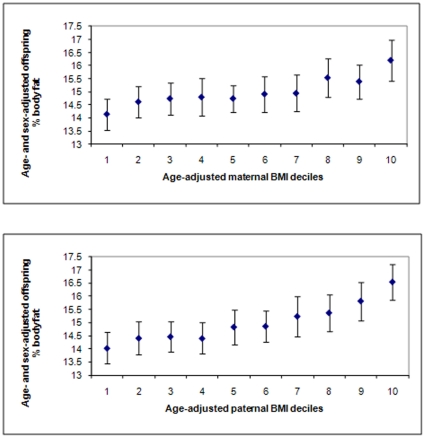
Offspring percentage body fat according to deciles of maternal (top panel) and paternal (bottom panel) body mass index BMI (based on 12,181 parent-offspring pairs available). Values are mean and 95% CIs.

We present age- and sex-adjusted offspring mean BMI, waist circumference and percentage body fatness by maternal and paternal deciles of their BMI (figures 1 – 3). We used multivariable linear regression to examine associations of parental BMI and parental overweight/obesity versus normal weight with offspring mean BMI, waist circumference, percent body fat and skinfold thicknesses (continuous variables) and multivariable logistic regression to examine these same associations with the binary offspring outcomes. In the basic models we controlled for trial arm, maternal and paternal age and child's sex and age. In subsequent models we adjusted for (i) potential confounding by urban vs rural residence and by family socioeconomic position (occupational social class, parental education), maternal and paternal smoking, and total number of siblings and (ii) the other parent's measurement.

Robust standard errors were used to compute confidence intervals and p-values to account for possible non-independence of measurements by hospital/polyclinic site (clustering). The study trial arm was included as a dummy variable in all regression models, as is conventional when randomised trials are used as cohort studies. However, the intervention (trial arm) was not associated with any of the measures of parental or childhood adiposity at 6.5 years.[19] Associations without adjustment for trial arm were essentially the same as those presented. F-statistics were computed to determine statistical evidence of a difference between maternal and paternal BMI associations with offspring continuous outcomes and chi-square tests were computed to examine differences with binary outcomes. In all multivariable analyses, only those children and parents with complete data on all covariables in any model were included. The possible impact of non-paternity on any weaker paternal (compared to maternal) associations was examined using Steer's correction of the Clemon's method for different assumed levels of non-paternity up to 20%.[24] In sensitivity analyses we also compared analyses: (a) when mothers reported parental anthropometry versus when fathers reported these measures; (b) when the child was the first born versus second or greater born; (c) when the mother was younger or older than 25 at the time of birth of her child. In these sensitivity analyses we repeated the fully adjusted multivariable associations within the two strata and compared whether they looked similar and computed likelihood ratio tests of interaction between parental BMI and the stratifying characteristic.

All analyses were conducted using Stata version 10.0 (Stata Corporation, Texas 2007).

## Results

Table S1 shows the characteristics of the trios included in this study. Overall 38% of the mothers, 52% of fathers, 10% of the daughters and 9% of the sons were overweight or obese (as defined by BMI thresholds). Mean waist circumference, percent body fat and skinfold thickness measurements were similar in daughters and sons.

Table 1 shows age standardised correlation coefficients between all parental and offspring anthropometric measurements. Mothers' and fathers' BMIs were weakly positively correlated with each other (Pearson's coefficient  = 0.18). All anthropometric measurements in the children assessed at age 6.5 years were moderately to strongly positively correlated with each other (coefficients ranging from 0.48 to 0.96). Birth weight was weakly positively correlated with all later offspring adiposity measurements (0.07 to 0.18). Maternal and paternal BMI were each weakly correlated with both daughters' and sons' anthropometric measurements at age 6.5 (0.09 to 0.21), with no clear pattern of differences between maternal and paternal associations or between associations with daughters and sons. Correlations with birth weight were stronger for mother's BMI than father's BMI (0.14 versus 0.08 for daughters and 0.14 versus 0.05 for sons).

**Table 1:** Age-standardised Pearson's correlations between parents and offspring anthropometry in daughters and sons. N = 5,869 daughters and 6,312 sons.

Daughters								
	Maternal BMI	Paternal BMI	Own birth weight	Own BMI @ 6.5 yrs	Own waist @ 6.5 yrs	Own percent fat @ 6.5 yrs	Own triceps skinfold @ 6.5 yrs	Own sub-scap skinfold @ 6.5 yrs
Maternal BMI	1.00							
Paternal BMI	0.18	1.00						
Birth weight	0.14	0.08	1.00					
Own BMI @ 6.5 yrs	0.21	0.17	0.18	1.00				
Own waist @ 6.5 yrs	0.16	0.15	0.17	0.73	1.00			
Own percent fat @ 6.5 yrs	0.13	0.15	0.10	0.65	0.58	1.00		
Own triceps skinfold @ 6.5 yrs	0.10	0.14	0.09	0.57	0.50	0.95	1.00	
Own sub-scap skinfold @ 6.5 yrs	0.16	0.13	0.08	0.67	0.61	0.83	0.66	1.00

Sons								
	Maternal BMI	Paternal BMI	Own birth weight	Own BMI @ 6.5 yrs	Own waist @ 6.5 yrs	Own percent fat @ 6.5 yrs	Own triceps skinfold @ 6.5 yrs	Own sub-scap skinfold @ 6.5 yrs
Maternal BMI	1.00							
Paternal BMI	0.17	1.00						
Birth weight	0.14	0.05	1.00					
Own BMI @ 6.5 yrs	0.18	0.21	0.16	1.00				
Own waist @ 6.5 yrs	0.14	0.16	0.16	0.71	1.00			
Own percent fat @ 6.5 yrs	0.11	0.15	0.09	0.61	0.55	1.00		
Own triceps skinfold @ 6.5 yrs	0.09	0.12	0.07	0.54	0.48	0.96	1.00	
Own sub-scap skinfold @ 6.5 yrs	0.12	0.16	0.09	0.63	0.58	0.83	0.66	1.00

All p-values for these coefficients <0.001.

In the multivariable regression analyses there was no strong or consistent statistical evidence for differences in associations between daughters and sons (all p-values > = 0.1 with few exceptions). There was some statistical evidence that mothers' BMI was more strongly associated with daughters' BMI than it was with sons' BMI, but the magnitude of the difference was small (mean difference in daughters' BMI per 1standard deviation (SD) mothers' BMI 0.21SD (95% CI: 0.18, 0.24) and in sons' BMI 0.17SD (0.13, 0.20), p for interaction  = 0.07; when offspring outcome was dichotomised as overweight/obese versus normal p for interaction  = 0.14), and that fathers' BMI was more strongly associated with sons' BMI than with daughters' BMI (mean difference per 1SD fathers BMI in daughters 0.14SD (95% CI: 0.12, 0.17) and in sons 0.18SD (0.14, 0.23), p for interaction  = 0.05 for these analyses and 0.09 when offspring BMI dichotomised as overweight/obese versus normal). There was also some evidence that mother's BMI was more strongly associated with daughter's than with sons'subscapular skinfold thickness (mean difference per 1SD mothers BMI in daughters 0.15SD (95%CI: 0.12, 0.19) and in sons 0.12SD (0.08, 0.15), p for interaction  = 0.07), but again the magnitude of the difference was small. These interactions represent 10% of the total number (40) examined and thus at the 0.1 level of significance differ little from what would be expected (10%) and could have arisen by chance. The main results comparing mothers' and fathers' associations with offspring outcomes did not change with the addition of gender interaction terms. All results for the remaining analyses are therefore presented with daughters and sons combined.

Figures 1 to [Fig pone-0014607-g002] 3 show mean age- and sex-adjusted offspring BMI, waist circumference and percent fat mass, respectively, by deciles of mothers' and fathers' BMI. For all offspring adiposity measurements, these graphs show very similar positive graded associations of increasing offspring adiposity with increasing parental decile across the entire distribution of both maternal and paternal BMI.

Multivariable associations are presented only for those trios with complete data on all covariables included in any model: 11,353 (93% of the eligible participants for this study). Table 2 shows the multivariable associations of maternal and paternal BMI (and overweight/obesity) with offspring mean BMI, waist circumference, percent fat mass and triceps and subscapular skinfolds. Maternal and paternal BMI were positively associated with all measurements of offspring adiposity. There was no strong statistical evidence that any of these associations differed in magnitude in mothers and fathers (all p-values > = 0.2).

**Table 2:** Multivariable associations of parental BMI with offspring anthropometry all assessed at age 6.5 years. N = 11,353 with complete data on all covariables.

Exposure		Mean difference in offspring outcomes (in SD units) by exposure (95%CI)									
		BMI		Waist		%Fat		Triceps SF		Subscapular SF	
**Maternal BMI per SD (1 SD = 4.4 kg.m^2^)**	**M1**	0.20	(0.17,0.23)	0.16	(0.13,0.18)	0.12	(0.09,0.15)	0.10	(0.06,0.13)	0.14	(0.11,0.17)
	**M2**	0.21	(0.18,0.25)	0.17	(0.15,0.20)	0.14	(0.11,0.17)	0.11	(0.09,0.14)	0.16	(0.13,0.19)
	**M3**	0.19	(0.16,0.21)	0.15	(0.13,0.17)	0.12	(0.09,0.14)	0.09	(0.07,0.12)	0.13	(0.11,0.16)
**Maternal overweight/obesity vs not**	**M1**	0.32	(0.26,0.37)	0.25	(0.19,0.30)	0.20	(0.13,0.26)	0.15	(0.09,0.22)	0.23	(0.18,0.28)
	**M2**	0.34	(0.29,0.40)	0.27	(0.22,0.33)	0.23	(0.18,0.29)	0.19	(0.13,0.25)	0.26	(0.21,0.31)
	**M3**	0.30	(0.25,0.35)	0.24	(0.19,0.29)	0.20	(0.14,0.25)	0.16	(0.11,0.21)	0.23	(0.17,0.28)
**Paternal BMI per SD (1 SD = 3.3 kg.m^2^)**	**M1**	0.19	(0.17,0.22)	0.16	(0.14,0.18)	0.16	(0.13,0.18)	0.14	(0.11,0.17)	0.16	(0.13,0.18)
	**M2**	0.20	(0.17,0.22)	0.16	(0.14,0.18)	0.15	(0.12,0.18)	0.13	(0.10,0.16)	0.15	(0.13,0.18)
	**M3**	0.16	(0.14,0.19)	0.13	(0.11,0.15)	0.13	(0.10,0.16)	0.12	(0.09,0.14)	0.13	(0.10,0.16)
**Paternal overweight/obesity vs not**	**M1**	0.30	(0.26,0.34)	0.24	(0.19,0.29)	0.24	(0.18,0.30)	0.21	(0.15,0.27)	0.24	(0.19,0.30)
	**M2**	0.31	(0.26,0.35)	0.24	(0.19,0.29)	0.24	(0.18,0.30)	0.20	(0.14,0.26)	0.24	(0.18,0.30)
	**M3**	0.26	(0.21,0.30)	0.20	(0.16,0.25)	0.21	(0.15,0.27)	0.18	(0.12,0.23)	0.20	(0.15,0.26)

SD: standard deviation; CI: confidence interval; BMI: Body Mass index; SF: Skinfold; Subscap.: Subscapular.

M1: Model 1: adjusted for randomisation arm, sex of offspring, age of offspring, age of parent.

M2: Model 2: as model 1 plus adjustment for potential confounding by occupational social class, parental education, rural vs urban, number of siblings, maternal smoking, paternal smoking.

M3: Model 3: as model 2 plus mutual adjustment of maternal and paternal BMI.

Table 3 shows equivalent multivariable associations for offspring outcomes dichotomised to represent those at the extreme overweight/obese end of the distribution. These showed similar findings to those in table 3 for continuous outcomes, with no strong evidence of differences in association between maternal and paternal BMI or overweight/obesity with any offspring anthropometry outcome (all p-values > = 0.3). Mothers and fathers who were overweight or obese were more likely to have overweight or obese offspring at age 6.5 years using any of the measurements of childhood adiposity. When we examined for the possible effects of non-paternity, results were essentially unchanged from those presented in tables 2 and 3 in sensitivity analyses assuming possible non-paternity up to levels of 20% (results available from authors on request).

**Table 3:** Multivariable associations of parental BMI with offspring anthropometry as binary outcomes all assessed at age 6.5 years. N = 11,353 with complete data on all covariables.

Exposure		Odds ratio for offspring outcomes by exposure (95%CI)									
		Overweight/obese		Large waist		High % fat		Large Triceps SF		Large subscap. SF	
**Maternal BMI per SD (1 SD = 4.4 kg.m^2^)**	**M1**	1.49	(1.40,1.58)	1.42	(1.33,1.51)	1.30	(1.20,1.40)	1.25	(1.16,1.36)	1.35	(1.24,1.46)
	**M2**	1.56	(1.46,1.65)	1.48	(1.39,1.57)	1.36	(1.26,1.46)	1.31	(1.22,1.41)	1.40	(1.28,1.53)
	**M3**	1.48	(1.39,1.57)	1.41	(1.32,1.49)	1.29	(1.21,1.39)	1.26	(1.17,1.35)	1.33	(1.22,1.45)
**Maternal overweight/obesity vs not**	**M1**	2.02	(1.77,2.31)	1.92	(1.65,2.22)	1.57	(1.36,1.83)	1.50	(1.28,1.76)	1.71	(1.44,2.04)
	**M2**	2.22	(1.94,2.54)	2.08	(1.82,2.39)	1.74	(1.49,2.01)	1.65	(1.43,1.90)	1.86	(1.54,2.24)
	**M3**	2.03	(1.77,2.31)	1.91	(1.67,2.18)	1.58	(1.36,1.84)	1.52	(1.33,1.75)	1.69	(1.40,2.03)
**Paternal BMI per SD (1 SD = 3.3 kg.m^2^)**	**M1**	1.53	(1.46,1.61)	1.47	(1.39,1.54)	1.42	(1.33,1.51)	1.37	(1.28,1.45)	1.45	(1.37,1.54)
	**M2**	1.53	(1.46,1.62)	1.46	(1.38,1.54)	1.41	(1.33,1.50)	1.36	(1.27,1.44)	1.45	(1.36,1.54)
	**M3**	1.45	(1.38,1.53)	1.39	(1.32,1.46)	1.35	(1.28,1.43)	1.31	(1.23,1.39)	1.39	(1.31,1.47)
**Paternal overweight/obesity vs not**	**M1**	2.00	(1.76,2.28)	1.91	(1.69,2.16)	1.89	(1.61,2.23)	1.72	(1.45,2.04)	1.97	(1.68,2.30)
	**M2**	2.02	(1.77,2.30)	1.91	(1.69,2.17)	1.89	(1.61,2.23)	1.72	(1.44,2.04)	1.97	(1.67,2.33)
	**M3**	1.81	(1.58,2.07)	1.72	(1.53,1.95)	1.76	(1.49,2.07)	1.60	(1.35,1.91)	1.81	(1.53,2.15)

CI: confidence interval; SF: Skinfold; Subscap.: Subscapular.

M1: Model 1: adjusted for randomisation arm, sex of offspring, age of offspring, age of parent.

M2: Model 2: as model 1 plus adjustment for potential confound by occupational social class, parental education, rural vs urban, number of siblings, maternal smoking, paternal smoking.

M3: Model 3: as model 2 plus mutual adjustment of maternal and paternal BMI.

Repeating all analyses with adjustment for whether the mother or father reported parental weight and height did not alter the findings presented here and results were the identical to those presented in table 2 and 3 if analyses were restricted just to participants where mothers had reported both parents' weight and height. For example, the fully adjusted (equivalent of model 3) association of maternal and paternal BMI with offspring BMI in the N = 10410 participants with maternal report of weight and height for both parents were 0.18 (0.15, 0.22) and 0.16 (0.14, 0.19), respectively. In the small sub-group (N = 626) for whom fathers reported parental weight and height associations tended to be slightly stronger for both parents (for example equivalent results to those in the previous sentence were 0.23 (0.15, 0.31) and 0.21 (0.15, 0.27)) but the associations remained similar to each other in this subgroup (i.e. mother-offspring associations remained similar to father-offspring associations) and there was no strong statistical evidence that any associations differed from those with mothers reporting (all p-values for difference >0.7). When we repeated analyses stratified by whether the index child was the first born or second or greater and by parental age (less than 25 years versus > = 25 at birth of child) the results were essentially identical in each strata (results available from authors on request).

## Discussion

Ours is one of the largest studies to date to examine the association between parental and offspring adiposity. In this population of families from Belarus, we found no evidence that associations of parental BMI, or categories of overweight/obese versus normal weight, with offspring BMI, waist circumference, skinfolds or derived percent fat mass were stronger in mothers than in fathers. Our findings do not support a greater effect of mothers (vs fathers) via mothers having more influence on their offspring diet or other adiposity related behaviours than fathers.

Since measurements of parental adiposity were obtained 6.5 years after the index pregnancy the extent to which we can explore specific maternal effects due to developmental overnutrition are limited. Repeat measurements of BMI in adulthood, including amongst women between pregnancies,[25]; [26] are strongly correlated. Therefore we expect that women (and their partners) would be ranked similarly by their BMI assessed 6.5 years post-pregnancy as during the index pregnancy, making our BMI measurements reasonable proxies for pre-pregnancy BMI. This is supported by our findings that parental BMI measured 6.5 years post pregnancy have similar magnitudes of association to offspring birthweight, including a stronger maternal than paternal association, to those found in cohorts in which parental BMI was measured pre- or in early pregnancy.[9] However, BMI tends to increase with increasing adult age, including between pregnancies,[26] and therefore more parents are likely to have been classified as overweight/obese in our study than they would had we used BMI assessed at the time of pregnancy, meaning that our results with parental BMI as a binary exposure may be biased as a test of the developmental overnutrition hypothesis. When we examined associations stratified by whether the child was a first born or higher order and by parental age results did not differ. Thus, our findings provide some evidence that suggests developmental overnutrition via greater maternal adiposity may not be particularly important in this population.

We further aimed to see whether we replicated findings from a previous small study suggesting that mother's adiposity was more specifically associated with daughters adiposity and father's with sons.[15] However, we found no strong evidence for such gender specific associations in our very large study, which is consistent with findings from a recent UK study of 4654 trios.[27] The authors of the initial report finding gender assortment in parental-offspring associations argued that this suggested genetic factors were unlikely to be important in familial clustering of adiposity. Our findings on a much larger sample argue against this.

Two previous large studies in East Asian populations reported larger maternal than paternal associations, but both only examined these associations with categorical exposures and outcomes.[6]; [7] In both studies the proportion of fathers in the obese category was considerably greater than the proportion of mothers defined as obese. Thus, the greater maternal effect may be in part driven by a ‘statistical’ artefact because of the obese mothers being at a more extreme end of the distribution of BMI than the obese fathers. Personal communication from the authors of one of these studies[7] suggests that on the continuous scale, maternal-offspring BMI associations (regression coefficient of maternal BMI standard deviation (z) score on offspring BMI z-score: 0.68 (95%CI: 0.40, 0.87) were stronger than paternal offspring-BMI associations (0.48 (0.30, 0.66)), but with only weak statistical evidence for a difference (p-value for difference  = 0.1) (personal communication Dr Connie Hui, Hong Kong). In an Australian birth cohort maternal-offspring BMI associations were stronger than paternal-offspring associations, with this finding remaining robust in sensitivity analyses accounting for non-paternity of up to 20%.[8] Similarly in a UK birth cohort maternal BMI was more strongly associated with offspring DXA determined fat mass than was paternal BMI, even after taking account of possible non-paternity[9] (though parental associations with offspring BMI in that cohort were the same[13]). Lastly, in a German cohort maternal-offspring BMI were more strongly associated than paternal-offspring BMI, though possible effects of non-paternity were not examined in that study.[10] All three of these studies had considerably smaller sample sizes (all 3000–4000) than our study and in all three the magnitude of parental association differences, whilst statistically robust, were small, all being in the region of 0.1SD difference in offspring BMI/fatmass comparing the association of 1SD maternal to paternal BMI. Our results are consistent with a previous large UK birth cohort that found no differences in associations of maternal BMI with offspring BMI compared to paternal-offspring associations[11] and with other smaller studies.[12]; [14] They are also consistent with a recent publication of a second large study (similar in size to the one presented here N = 15, 976) from India, which found similar magnitudes of maternal and paternal BMI with risk of underweight and stunting in offspring[28], although findings with respect to offspring BMI in the normal range were not presented in that study.

The similar magnitude of association for mothers and fathers, and the lack of gender assortment, are consistent with both genetic and familial lifestyle mechanisms explaining familiar clustering of adiposity. The population level obesity epidemic is unlikely to be driven by genetic factors and at an individual level genetic manipulation is currently not possible. Moreover, the mechanisms for obesity prevention (via diet and physical activity) are the same for those with or without a genetic predisposition to greater adiposity. Thus, our findings further highlight the importance of interventions aimed at the whole family for the prevention of childhood overweight/obesity.[29]

### Study strengths and limitations

The main strengths of this study are its large sample size and offspring outcomes that include BMI, waist circumference and percent fat mass derived from skinfold thickness. Parental weight and height were self-reported, and for the vast majority, mothers reported both their own weights and heights and those of the fathers. This could result in bias of associations with fathers' exposures towards equivalent associations in mothers if mothers tended to systematically bias reports of fathers anthropometry to be similar to their own size. However, the correlation between spousal BMI values is similar in this study to that of other studies where father's reported their own weight and height, suggesting that this is an unlikely source of bias. For example, in a general population UK birth cohort[9] and a general population Finnish children's cohort[14] in which mothers and fathers reported their own weight and height (rather than one parent, usually mother, reporting for both of them) correlation coefficients were 0.16 and 0.18, respectively, compared with 0.18 in this study. In the study from India described above were parental weight and height were measured correlations were slightly higher at 0.24,[28] which may reflect stronger assortative marriage patterns being evident in that society or the more accurate measurement of anthropometry compared with self-report. These findings do not suggest that greater correlation between spouses in our study is a result of mothers reporting their own and their husband's anthropometry. Although numbers with parental weight and height reported by fathers was small in our study, there was no evidence that associations in this group differed from those in the majority of the sample where mothers had reported weight and height for both parents.

We were able to examine associations with offspring outcomes only up to age 6.5 years, and because of the narrow age range in the study (75% of participants were aged 6.4 to 6.6 years) we were unable to examine whether associations strengthened with increasing offspring age in this population. There is some evidence that associations do amplify with age. For example, in the British 1958 birth cohort, maternal and paternal BMI correlations with sons' and daughters' BMI were all 0.15 or 0.16 at age 7 years but increased to 0.20 to 0.24 at age 33 years.[30] In childhood, correlations were identical for mothers' and fathers' exposures. For ages 23 and 33 there was some suggestion that mothers' BMI was more strongly related to daughters' BMI than was fathers' BMI (0.24 versus 0.15), but no parental difference was observed for sons' BMI. Thus, mothers may have a stronger influence on their daughters' BMI than do fathers' once they reach adulthood and long-term follow-up of our cohort into adulthood will be able to examine this further. That said, the differences between mothers and fathers are small (0.09 of a SD of offspring BMI per 1SD of parental BMI) and unlikely to be a major driver of the obesity epidemic.

Lastly, the population we have studied is relatively lean and healthy. Mean maternal BMI in this cohort was 24.5 k/m^2^ and only 12% of the mothers and 10% of the fathers were above BMI thresholds used to define obesity (though 38% and 52% respectively were either overweight or obese); only 2% each of daughters and sons were classified as obese (10% and 9% overweight or obese). These prevalences of overweight/obesity whilst not extremely low are lower than in contemporary Western populations. For example, in the US 62% of female adults, 71% of male adults and >30% of children are overweight/obese by the same criteria.[31]; [32] To be included in the study mothers had to have initiated breastfeeding in the immediate postnatal period. However, at the time of recruitment to this study 95% of Belarus mothers with healthy term babies did so and therefore failure to initiate breast feeding (which may be related to obesity) was not a major reason for exclusion and is unlikely to have been a source of selection bias. We are aware of only one previous publication of overweight/obesity prevalences in Belarus. Using information from the WHO database Jarosz and Rychlik reported the prevalence of overweight and obesity combined in adults aged 15 years or over to be 69.9% and 63.7% in females and males, respectively. These higher prevalences than in our cohort may reflect the much wider age range, including adolescents/young adults or the fact that the data were collected in 1985, whereas ours were collected between 2002 and 2005.

### Conclusions and implications

In this large study of families from Belarus, we have confirmed the findings from other populations of a graded linear association of BMI in both parents with offspring BMI, and have also shown that this association extends to offspring waist circumference and percent fat mass. The magnitude of the associations between maternal-offspring adiposity and paternal-offspring adiposity were similar and we found no evidence for gender assortment, suggesting that genetic and/or shared familial environment explain this family clustering. A number of previous studies have compared maternal-offspring to paternal-offspring adiposity associations as described above, and considering this previous evidence together with our large study, we would conclude that there is little evidence to support a difference in the association of maternal BMI with offspring adiposity compared with the association of paternal BMI with offspring adiposity. If a difference does exist, even at older offspring ages than investigated in this study, it is small in magnitude and unlikely to be a major driver of the obesity epidemic. In terms of potential long-term effects of developmental overnutrition, increasingly evidence from sibling studies does suggest a causal intrauterine effect of more extreme phenotypes – i.e. extreme obesity and diabetes in pregnancy – on future offspring adiposity, [33]–[35] but there is currently little evidence of a linear effect of greater maternal BMI during pregnancy across most of the normal range having a long term programming effect on offspring BMI. In public health terms our findings suggest that interventions aimed at reducing mean BMI that were targeted specifically or solely at women of reproductive age would not have a disproportional beneficial influence for the whole population. Interventions aimed at changing overall family lifestyle are likely to be important for population level obesity prevention.

## Supporting Information

Table S1 Supplementary web material.
(0.7 MB DOC)
