# Supplementary material for: Familial Associations of Adiposity: Findings from a Cross-Sectional Study of 12,181 Parental-Offspring Trios from Belarus
Source: PLoS One. 2011 Jan 27;6(1):e14607. doi: 10.1371/journal.pone.0014607 (PMC3029263; doi:10.1371/journal.pone.0014607)
Supplement: Table S1 — Supplementary web material. (0.7 MB DOC) [file pone.0014607.s001.doc]

# SUPPLEMENTARY WEB MATERIAL FOR:

# Familial associations of adiposity: findings from a cross-sectional study of 12,181 parental-offspring trios from Belarus.

# Rita Patel,^1^ Richard M Martin,^1,2^ Michael S. Kramer,^3,4^ Emily Oken,^5^ Natalia Bogdanovich,^6^ Lidia Matush,^6^ George Davey Smith,^1,2^ Debbie A Lawlor.^1,2^

^1^ School of Social and Community Medicine, University of Bristol, Bristol, UK

^2^ Medical Research Council Centre for Causal Analyses in Translational Epidemiology, University of Bristol, Bristol, UK

^3^ Department of Pediatrics, McGill University Faculty of Medicine, Montreal, Canada

^4^ Department of Epidemiology and Biostatistics, McGill University Faculty of Medicine, Montreal, Canada

^5^ Department of Population Medicine, Harvard Medical School and Harvard Pilgrim Health Care Institute, Boston, USA

^6^ The National Research and Applied Medicine Mother and Child Centre, Minsk, Belarus

**Supplementary Webtable 1: Characteristics of participants included in analyses. N = 12,181**

|  |  | **Daughters N=5,869** | |  | **Sons**  **N=6,312** | |
| --- | --- | --- | --- | --- | --- | --- |
| **Characteristic** | **Category or scale** | **N (%), or mean (SD)** | |  | **N (%), or mean (SD)** | |
| Maternal BMI | Mean kg/m^2^ | 24.5 | (4.4) |  | 24.5 | (4.4) |
| Maternal overweight/obese | BMI < 25 | 3656 | (62%) |  | 3945 | (63%) |
|  | BMI >= 25 | 2213 | (38%) |  | 2367 | (38%) |
| Paternal BMI | Mean kg/m^2^ | 25.7 | (3.3) |  | 25.7 | (3.3) |
| Paternal overweight/obese | BMI < 25 | 2789 | (48%) |  | 2933 | (46%) |
|  | BMI >= 25 | 3080 | (52%) |  | 3379 | (54%) |
| Offspring birth weight | Mean g | 3379.2 | (404.2) |  | 3516.2 | (424.3) |
| Offspring BMI | Mean kg/m^2^ | 15.4 | (1.7) |  | 15.7 | (1.6) |
| Offspring Overweight/obese | Below IOTF threshold | 5298 | (90%) |  | 5755 | (91%) |
|  | Above IOTF threshold | 571 | (10%) |  | 557 | (9%) |
| Offspring waist circumference | Mean mm | 53.9 | (4.5) |  | 54.8 | (4.2) |
| Offspring large waist | < 90^th^ percentile | 5302 | (90%) |  | 5769 | (91%) |
|  | > = 90^th^ percentile | 567 | (10%) |  | 543 | (9%) |
| Offspring percent fat | Mean % | 15.6 | (4.7) |  | 14.4 | (4.7) |
| Offspring high % fat | < 90^th^ percentile | 5335 | (91%) |  | 5734 | (91%) |
|  | > = 90^th^ percentile | 534 | (9%) |  | 578 | (9%) |
| Offspring triceps skinfold | Mean mm | 10.5 | (3.9) |  | 9.4 | (3.6) |
| Offspring high triceps skinfold | < 90^th^ percentile | 5385 | (92%) |  | 5769 | (91%) |
|  | > = 90^th^ percentile | 484 | (8%) |  | 543 | (9%) |
| Offspring subscapular skinfold | Mean mm | 6.2 | (2.5) |  | 5.6 | (2.1) |
| Offspring high subscapular skinfold | < 90^th^ percentile | 5364 | (91%) |  | 5879 | (93%) |
|  | > = 90^th^ percentile | 505 | (9%) |  | 433 | (7%) |
| Offspring age at follow-up | Mean years | 6.6 | (0.3) |  | 6.6 | (0.3) |
| Maternal age at birth of child | Mean years | 25.1 | (4.9) |  | 25.1 | (4.9) |
| Paternal age at birth of child | Mean years | 27.5 | (5.1) |  | 27.5 | (5.1) |
| Head of household social class | Manual | 2379 | (41%) |  | 2561 | (41%) |
|  | Non-manual | 3216 | (55%) |  | 3427 | (54%) |
| Maternal education | Initial, incomplete or common secondary only | 1991 | (34%) |  | 2171 | (34%) |
|  | Advanced secondary or partial university | 3052 | (52%) |  | 3271 | (52%) |
|  | Completed university | 826 | (14%) |  | 870 | (14%) |
| Paternal education | Initial, incomplete or common secondary only | 2291 | (39%) |  | 2464 | (39%) |
|  | Advanced secondary or partial university | 2802 | (48%) |  | 2988 | (47%) |
|  | Completed university | 767 | (13%) |  | 848 | (13%) |
| Residence | Rural | 2678 | (46%) |  | 2841 | (45%) |
|  | Urban | 3191 | (54%) |  | 3471 | (55%) |
| Number of siblings at time of 6.5 year follow-up | 0 | 2145 | (37%) |  | 2254 | (36%) |
|  | 1 | 2923 | (50%) |  | 3140 | (50%) |
|  | 2 | 538 | (9%) |  | 617 | (10%) |
|  | 3 | 148 | (3%) |  | 144 | (2%) |
|  | > = 4 | 115 | (2%) |  | 157 | (2%) |
| Maternal smoking in pregnancy | No | 5786 | (99%) |  | 6201 | (98%) |
|  | Yes | 83 | (1%) |  | 111 | (2%) |
| Maternal smoking at time of 6.5 year follow-up | No | 5245 | (89%) |  | 5671 | (90%) |
|  | Yes | 612 | (10%) |  | 626 | (10%) |
| Paternal smoking at time of 6.5 year follow-up | No | 2127 | (36%) |  | 2222 | (35%) |
|  | Yes | 3633 | (62%) |  | 3975 | (63%) |
| Randomised to breast feeding promotion | No | 2898 | (49%) |  | 3154 | (50%) |
|  | Yes | 2971 | (51%) |  | 3158 | (50%) |
